# Snapshot: Implications for mTOR in Aging-related Ischemia/Reperfusion Injury

**DOI:** 10.14336/AD.2018.0501

**Published:** 2019-02-01

**Authors:** Dong Liu, Liqun Xu, Xiaoyan Zhang, Changhong Shi, Shubin Qiao, Zhiqiang Ma, Jiansong Yuan

**Affiliations:** ^1^State Key Laboratory of Cardiovascular Disease, Fuwai Hospital, National Center for Cardiovascular Diseases, Chinese Academy of Medical Sciences and Peking Union Medical College, Beijing, 100037, China.; ^2^Department of Thoracic Surgery, Tangdu Hospital, The Fourth Military Medical University, 1 Xinsi Road, Xi’an 710038, China.; ^3^Cadet group 3, School of Basic Medical Sciences, The Fourth Military Medical University, Xi’an 710032, China.; ^4^Laboratory Animal Center, The Fourth Military Medical University, Xi’an 710032, China

**Keywords:** Ischemia/reperfusion injury, Aging, mTOR, Autophagy

## Abstract

Aging may aggravate the damage and dysfunction of different components of multiorgan and thus increasing multiorgan ischemia/reperfusion (IR) injury. IR injury occurs in many organs and tissues, which is a major cause of morbidity and mortality worldwide. The kinase mammalian target of rapamycin (mTOR), an atypical serine/threonine protein kinase, involves in the pathophysiological process of IR injury. In this review, we first briefly introduce the molecular features of mTOR, the association between mTOR and aging, and especially its role on autophagy. Special focus is placed on the roles of mTOR during ischemic and IR injury. We then clarify the association between mTOR and conditioning phenomena. Following this background, we expand our discussion to potential future directions of research in this area. Collectively, information reviewed herein will serve as a comprehensive reference for the actions of mTOR in IR injury and may be significant for the design of future research and increase the potential of mTOR as a therapeutic target.

Aging is a complex and progressive process that involves physiological and metabolic deterioration in every organ and system [1]. It represents a triple threat for ischemia/reperfusion (IR) injury [2]. Not only does the incidence of ischemia increase with age, but the organs or tissues become more susceptible to ischemic damage and the protective interventions such as ischemic preconditioning (IPC) become less effective [2]. IR injury occurs when the blood supply to the tissue is blocked for minutes to hours (ischemia) and then restored (reperfusion) [3, 4]. Ischemia elicits tissue anoxia which is the basis of ischemic injury and primes the tissue for subsequent reperfusion damage [3]. IR injury affects many organs and tissues including heart [5, 6], brain [7-9], liver [10], kidney [11, 12], lung [13], skeletal muscles [14], and testes tissue [15], contributing to high morbidity and mortality worldwide. Numerous efforts have attempted to search for proper agents for the treatment of IR injury every year. Notably, the roles of the kinase mammalian target of rapamycin (mTOR) on IR injury get much attention in recent years and multiple novel mechanisms have been revealed [14, 16-18]. mTOR is an atypical serine/threonine protein kinase that belongs to the phosphoinositide 3 kinase (PI3K)-related kinase family and interacts with several proteins to form two distinct complexes named mTOR complex 1 (mTORC1) and 2 (mTORC2) [19]. mTOR plays a central role in regulating many fundamental cell processes in eukaryotic cell, from protein synthesis to autophagy, and deregulated mTOR signaling is implicated in the progression of cancer and diabetes, as well as the aging process [20]. Emerging evidence suggests that mTORC1 inhibition has positive effects on multiple age-related pathologies in rodents and, in some cases, humans [21]. Autophagy is a conserved and programmed catabolic process that degrades damaged proteins and organelles, and contributes significantly to the degree of IR injury [22], which indicates mTOR involving in the pathophysiological process of IR injury.

This article reviews the information available regarding the latest progress regarding the effects of mTOR in IR injury. We first briefly introduce the molecular features and functions of mTOR in IR injury, the association between mTOR and aging, and especially the mTOR related autophagy in the pathophysiology of IR injury. We then clarify the roles of mTOR during ischemic and IR injury. Special focus is placed on the signaling pathways of mTOR during IR injury. Additionally, we introduce the association between mTOR and conditioning phenomena. Finally, we discuss several novel potential directions for future research in this area. The information compiled, herein, may serve as a comprehensive reference for the activities of mTOR in IR and may be helpful for the design of future studies and for the future development of mTOR as a therapeutic target.

**Figure 1.**
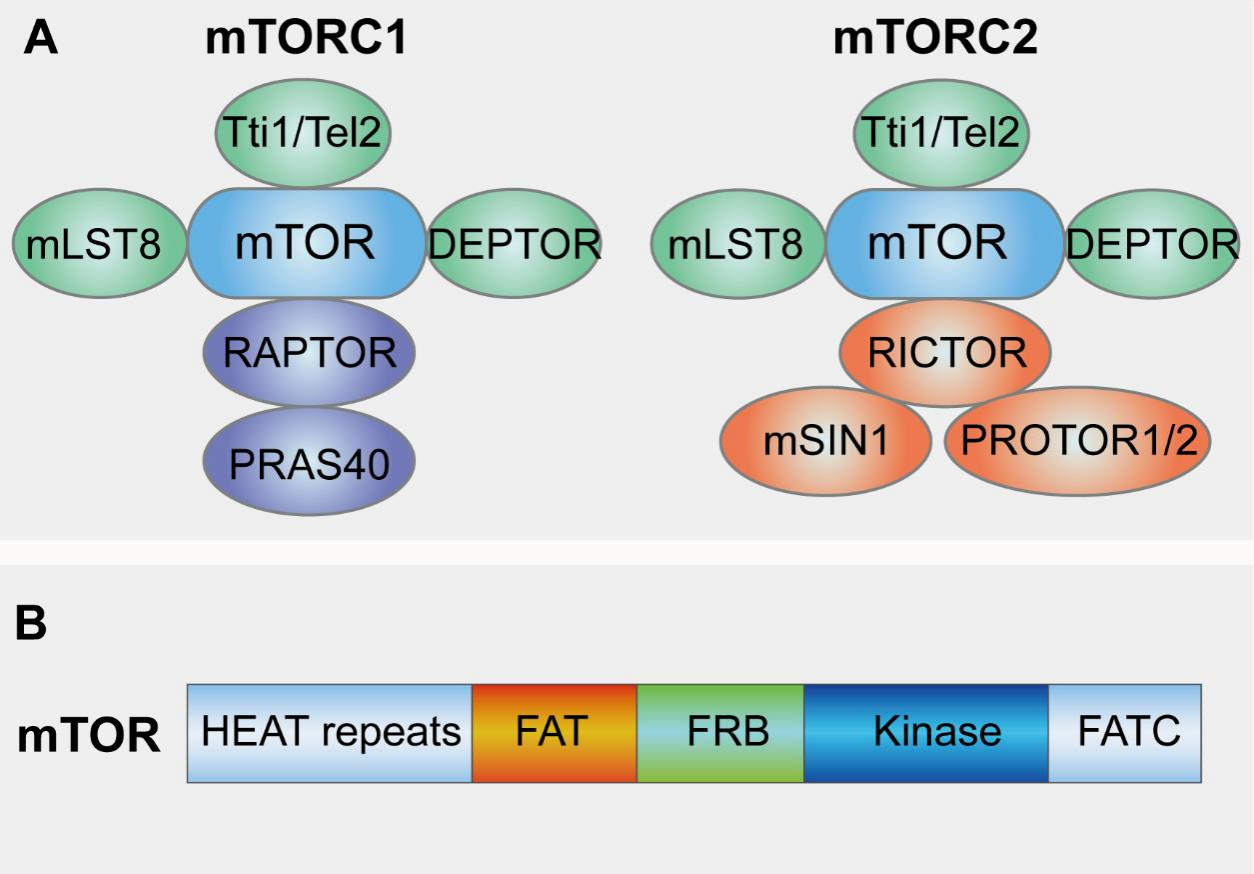
**Structural characteristics of mTOR and mTORC1/2**. (**A**) part illustrates the structure of mTORC1 and mTORC2. The mTOR kinase nucleates two distinct protein complexes termed mTORC1 and mTORC2. mTORC1 contains six known protein components: mTOR, regulatory protein associated with mTOR (Raptor), mammalian lethal with Sec13 protein 8 (mLST8), proline-rich Akt substrate of 40 kDa (PRAS40), DEP domain containing mTOR interacting protein (DEPTOR) and the Tti1/Tel2 complex. mTORC2 containing seven protein components constitutes mTOR, DEPTOR, mLST8, Tti1/Tel2 complex, Protor1/2 mammalian stress-activated protein kinase-interacting protein 1 (mSin1) and rapamycin insensitive companion of mTOR (Rictor). (**B**) This diagram depicts the structure of mTOR. mTOR are characterized by five distinct protein domains: FAT-carboxy terminal domain (FAT domain), FRAP-ATM-TTRAP domain (FATC domain), FKBP12-rapamycin binding domain (FRB domain), Huntingtin-Elongation factor 3-regulatory subunit A of PP2A-TOR1 repeats (HEAT repeats).

## General background on mTOR

### Foundations and structural characteristics of mTOR

As the name implies, the discovery of the mammalian target of rapamycin (mTOR) is intimately linked to the discovery of rapamycin [23]. mTOR is the target of a molecule named rapamycin or sirolimus, which is a macrolide produced by Streptomyces Hygroscopicus bacteria and that first gained attention because of its broad antiproliferative properties [24, 25]. Studies in the budding yeast Saccharomyces cerevisiae first identified the target of rapamycin genes TOR1 and TOR2 as genetic mediators of rapamycin’s growth inhibitory effects, and soon afterwards the mTOR protein was purified from mammalian cells and demonstrated to be the physical target of rapamycin [19, 21]. mTOR is an evolutionarily conserved serine/threonine protein kinase that regulates multiple cellular processes such as cell growth, cell cycle, cell survival, and autophagy [19]. mTOR forms two functional complexes, mTORC1 and mTORC2, the configuration of which is conserved from yeast to mammals [26]. mTORC1 contains six known protein components: mTOR, Raptor (regulatory protein associated with mTOR), and mLST8 (mammalian lethal with Sec13 protein 8) [27-29], PRAS40 (proline-rich Akt substrate of 40 kDa) [30, 31], DEPTOR (DEP domain containing mTOR interacting protein) [32] and the Tti1/Tel2 complex [33] (Fig. 1A). mTOR is characterized by five distinct protein domains: FAT-carboxy terminal domain (FAT domain), FRAP-ATM-TTRAP domain (FATC domain), FKBP12-rapamycin binding domain (FRB domain), Huntingtin-Elongation factor 3-regulatory subunit A of PP2A-TOR1 repeats (HEAT repeats) [20] (Fig. 1B). Raptor facilitates substrate recruitment to mTORC1 through binding to the TOR signaling (TOS) motif found on several canonical mTORC1 substrates [34, 35], and also plays a significant role in intracellular localization of mTORC1 in response to amino acid availability, which is an essential cellular cue form TORC1 activation [36]. mLST8 by contrast associates with the catalytic domain of mTORC1 and may stabilize the kinase activation loop [37], though genetic studies suggest it is dispensable for the essential functions of mTORC1 [38]. Like mTORC1, mTORC2 containing seven protein components also constitutes mTOR, DEPTOR [32], Tti1/Tel2 complex and mLST8. Instead of Raptor, mTORC2 contains Protor1/2 [39-41], mSin1 (mammalian stress-activated protein kinase-interacting protein 1 ) [42-44] and Rictor (rapamycin insensitive companion of mTOR) that is an unrelated protein that likely serves an analogous function [45, 46] (Fig. 1A). Rapamycin forms a complex with FK506-binding protein 12 (FKBP12) and as a complex inhibits mTORC1 via blocking its interaction with Raptor while mTORC2 is characterized by its insensitivity to acute rapamycin treatment [47]. Interestingly, although rapamycin-FKBP12 complexes do not directly bind or inhibit mTORC2, prolonged rapamycin treatment does abrogate mTORC2 signaling, likely due to the inability of rapamycin-bound mTOR to incorporate into new mTORC2 complexes or alters the mTORC1/C2 equilibrium resulting in reduced mTORC2 levels and impaired Akt signaling [48, 49].

### Activation of mTOR and signal transduction

**Figure 2.**
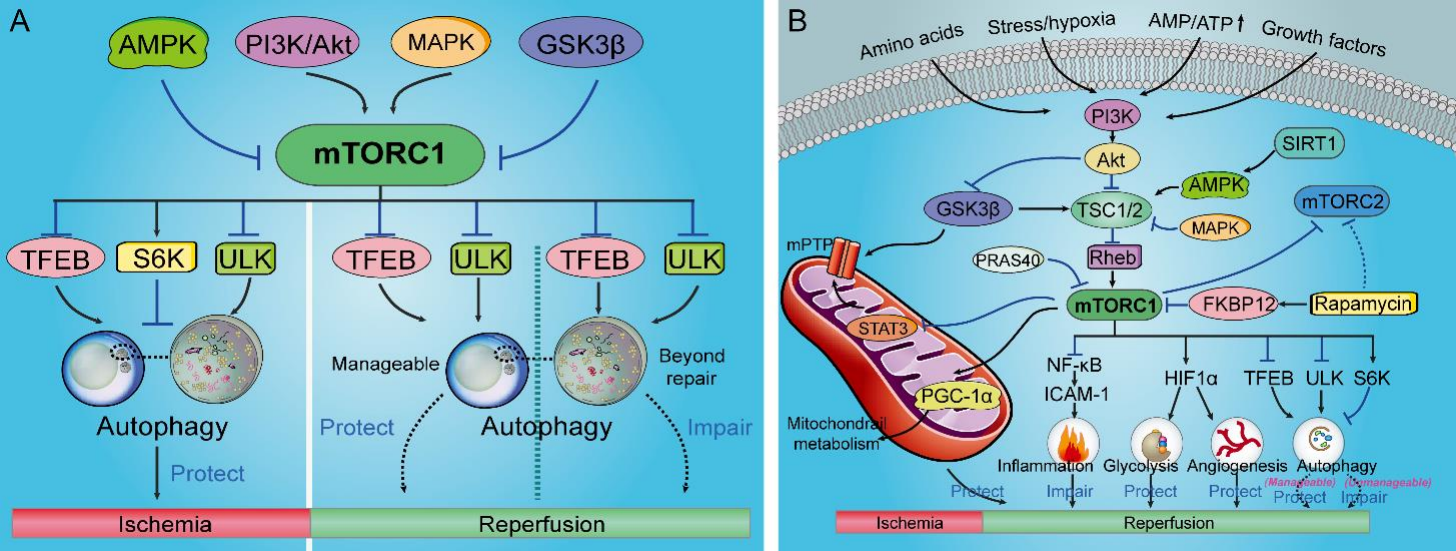
**mTORC1 related autophagy signaling in ischemic and ischemia/reperfusion injury and mTORC1/2 signaling pathways involved in IR injury**. (**A**) mTORC1 inhibition thus activating autophagy during ischemia protects against ischemia injury. However, the role of mTORC1 signaling and autophagy in reperfusion injury is complicated. Protective autophagy via suppression of mTORC1 can reduce reperfusion injury while excessive autophagy may increase the injurious effects of reperfusion. (**B**) The mTORC1/2 signaling pathways involved in IR injury. Abbreviations: 4E-BP1, eIF4E-binding protein-1; AMP, adenosine monophosphate; AMPK, adenosine monophosphate-activated protein kinase; Akt, protein kinase B; ATP, adenosine triphosphate; FKBP12, FK506-binding protein 12; GSK-3β, glycogen synthase kinase-3β; HIF-1α, transcription factor-1α; MAPK, mitogen-activated protein kinase; mPTP, mitochondrial permeability transition pore; mTORC, mammalian target of rapamycin complex; NF-κB, nuclear factor-κB; PGC-1α, peroxisome-proliferator-activated receptor coactivator-1α; PI3K, phosphoinositide 3 kinase; Rheb, Ras homolog enriched in brain; S6K, S6 kinase; STAT3, signal transducer and activator of transcription 3; TFEB, transcription factor EB; TSC, tuberous sclerosis protein; ULK, unc-51-like kinase.

The mTOR pathway integrates inputs from a variety of different classes of stimuli and recent studies show mTOR is activated after stimulation by Toll-like receptors (TLRs) [50, 51]. Much more is known about both the upstream regulation and downstream outputs of mTORC1 compared with mTORC2. mTORC1 is activated by growth factors and repressed by acid deprivation, hypoxia, energy stress, ER stress, genotoxic stress and adenosine monophosphate-activated protein kinase (AMPK), a key sensor of cellular energy status [19]. In response to these growth signals, mTORC1 phosphorylates and activates ribosomal protein S6 kinases (S6K1), which in turn promotes mRNA biogenesis and activates the protein translation process. In contrast, mTORC1 inhibits eukaryotic translation initiation factor 4E-binding protein 1 (4E-BP1) and allows the formation of the eIF4F complex that triggers cap-dependent translation [21, 52-54]. In addition, mTORC1 promotes glucose metabolism through the hypoxic response transcription factor-1α (HIF-1α) as well as regulates mitochondrial function and metabolism via the peroxisome-proliferator-activated receptor coactivator-1α (PGC-1α), promotes lipid biosynthesis and represses degradation through the autophagy pathway [21] (Fig. 2B). mTORC2 is activated by growth factor stimulated PI3K whereas it is relatively insensitive to nutrient deprivation, and little is known about the molecular mechanism of this activation other than that it involves mTORC2-ribosome association [19]. The ribosome has recently been shown to be a necessary factor for mTORC2 activity [55, 56], indicating that a cell’s capacity to sustain growth is related to mTORC2. Furthermore, mTORC1 seems to inhibit mTORC2 through phosphorylation of Rictor, suggesting that mTORC1 and mTORC2 are functionally interconnected [57]. mTORC2 is the upstream activating kinase for AGC (protein kinase A, protein kinase G, protein kinase C) kinase family members, including Akt which is an important oncoprotein that activates a broad anti-apoptotic programme for cell survival [58], various protein kinase C family members and serum/ glucocorticoid-regulated kinase 1 (SGK1) [59]. SGK1 has been shown to promote cardiomyocyte survival while inhibiting hypertrophy, whereas SGK1 chronic activation during heart failure is detrimental [57, 60]. mTORC2 also mediates actin cytoskeletal organization through the regulation of PKC-α and Ras homolog gene family member A [45], and a recent study found this function has significance in tumour cell motility, invasiveness and metastasis [54, 61].

### The association between mTOR and aging

Aging is a heterogeneous process with some individuals reaching advanced ages with perturbations to decline in protein synthesis [62, 63], increasing lipid profiles and in?ammatory processes [64]. Substantial evidence suggests that mTOR signaling is strongly implicated in the aging process of diverse organisms (e.g., yeast, worms, ?ies and mammals), and mTOR pathway is responsive to changes in energy status, nutrient availability, in?ammatory changes and DNA damage [21, 65-67]. This is supported by evidence, still controversial, that mTORC1 signaling may be aberrantly upregulated during the aging process [68], and mTORC1 signaling is hyperactivated in a wide range of diseases, including many not directly linked to aging [65]. Furthermore, in?ammatory signals are known to activate mTORC1, and the enhanced chronic in?ammation that accompanies aging may lead to high basal mTORC1 activation, in turn driving pathologic processes [65]. Age-related alterations in heart, liver, adrenal glands, endometrium and tendon, as well as the decline in spontaneous activity, all occur more slowly in rapamycin-treated mice [69], which indicating that inhibition of mTOR pathway extends lifespan in model organisms and confers protection against a growing list of age-related pathologies [21].

### mTOR related autophagy in the pathophysiology of IR injury

As mentioned above, mTOR is a key inhibitor of autophagy and repression of mTOR promotes autophagic activity [22]. Autophagy (or self-eating) was described as a lysosomal degradation pathway that removes protein aggregates and damaged cytoplasmic constituents, thereby maintaining intracellular homeostasis under various physiological and pathological conditions [70]. Evidence suggests that autophagic degradation declines with age, and it has been proposed that this leads to an accumulation of damage, such as protein aggregates and degenerate mitochondria, that contributes to age-related cellular dysfunction [71]. mTORC1 regulates autophagy which is regulated during energy deprivation and ischemia both at transcriptional and post-translational levels [72]. mTOR significantly activites p70S6K1 and inhibits the transcription factor EB (TFEB) that can induce autophagy through the upregulation of autophagic proteins such as Atg7 [73-75] (Fig. 2A). It has been demonstrated that mTORC1 phosphorylates the autophagic protein unc-51-like kinase 1/2 (ULK1/2), thereby inhibiting the macrocomplex ULK1/autophagy-related gene 13 (Atg13)/focal adhesion kinase family interacting protein-200 that promotes autophagosome formation [76]. Furthermore, mTORC1 activation inhibits the expression of autophagic proteins, particularly Atg7 that is vital for the initiation of the autophagic process [77]. Aberrant regulation of autophagy has been linked to several diseases of aging, including diabetes, cardiovascular diseases and neurodegenerative diseases, and it seems likely that enhanced autophagy underlies many of the beneficial effects of mTORC1 inhibition in these disease models [78].

mTORC1 is inhibited during energy deprivation and ischemia, and mTORC1 inhibition preserves the energy status through the reduction of cellular energy expenditure and activation of autophagy thus promotes survival (Fig. 2A). Stimulating autophagy via activation of AMPK thus inhibiting mTOR rescues ischemic injury and increases the viability of mesenchymal stem cells [14, 79]. However, the role of mTOR signaling and autophagy in reperfusion injury is still controversial. Inhibition of autophagy with a dominant negative inhibitor of Atg5 abolishes the infarct size reduction by IPC in HL-1 cells, indicating autophagy plays a protective role in myocardial IR injury [22, 80]. Ischemic postconditioning (IPostC) increases the expression of autophagy-related proteins, and 3-methyl-adenine, a pharmacological inhibitor of autophagy abrogates the infarct size reduction by IPostC [81]. However, another studies found that autophagy can also be deleterious during reperfusion [82, 83]. Inhibition of autophagy by pretreatment urocortin, an endogenous cardiac peptide reduced the percentage of myocytes death after IR [82]. Additionally, a combination of four active compounds alleviates cerebral IR injury in correlation with inhibition of autophagy and modulation of AMPK/mTOR and JNK pathways [83]. Autophagy is activated as an adaptive response to maintain the survival of cells under stressful conditions while if the cellular stress is not manageable and elicits damage that is beyond repair, the activation of autophagy may drive cells to die [22], indicating that whether autophagy acts as a protective mechanism or contributes to the injurious effects of IR injury in tissues or organs may depend on autophagy activation level and experimental context (Fig. 2A). Protective not excessive autophagy regulated via mTOR signaling will be a useful strategy for the treatment of IR injury.

**Figure 3.**
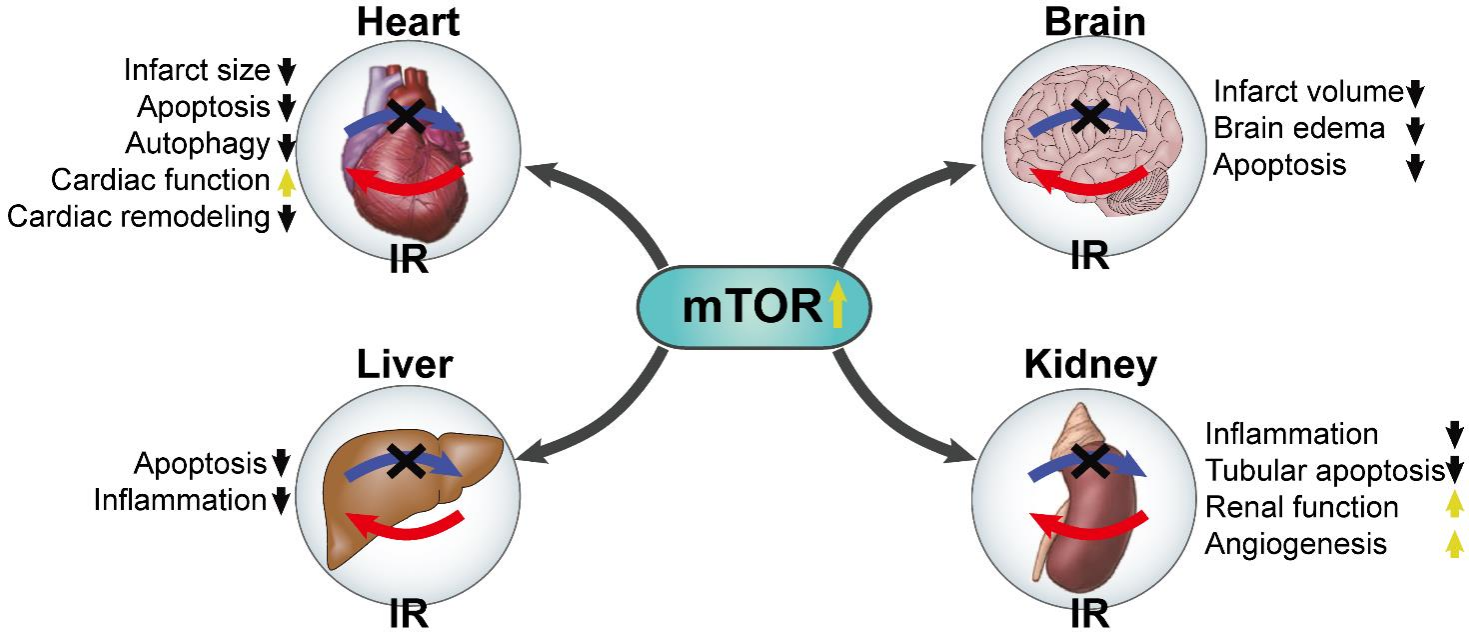
**The protective roles of mTOR against multiorgan IR injury**. The blue arrows with dark cross represent ischemia and the red arrows represent reperfusion.

## The deleterious role of mTOR during ischemic injury

The disease state of ischemia results from a hypoperfusion-induced insufficiency of oxidative metabolism due to inadequate blood circulation and the incidence of ischemia increase with age [2, 84]. Accumulating evidences indicate that mTOR is involved in ischemic injury and mTORC1 is inhibited during acute ischemia which preserves the energy status through the reduction of cellular energy expenditure and activation of autophagy and promotes survival [17, 72, 85]. Activation of mTOR during ischemia may lead to decreased autophagy and increased ischemic injury [86]. Rapamycin reduces myocardial infarction in the diabetic mice heart via inhibiting mTOR thus activating the Janus kinase 2 (JAK2) /signal transducer and activator of transcription 3 (STAT3) signaling pathway [87] (Fig. 2B). It recently demonstrated that mTORC1 was inhibited during ischemia through the inhibition of Ras homolog enriched in brain (Rheb) which directly binds and activates mTORC1 [22, 88, 89]. Mice with partial cardiac Rheb deletion display better cardiac function after experimental myocardial infarction and a reduction of infarct size as compared with control mice, indicating Rheb inhibition is beneficial and corroborating the protective effect of mTORC1 inhibition during acute ischemia [90]. Furthermore, ischemic injury of skeletal muscles is a common pathophysiology during peripheral vascular injury and surgeries [85, 91], which usually induces significant necrosis and apoptosis in the skeletal muscle cells. Endothelial mTORC1 deletion protects against hindlimb ischemic injury in diabetic mice possibly via activation of autophagy, attenuation of oxidative stress and alleviation of inflammation [85].

A reduction in cellular energy charge such as glucose deprivation, activates AMPK, a major energy-sensing kinase that is activated by an increase in the AMP/ATP ratio, hypoxia, and ROS production [92-94], which inhibits mTORC1 through phosphorylation and activation of tuberous sclerosis complex (TSC) indirectly as well as through the phosphorylation of Raptor directly [95, 96]. TSC is a heterotrimeric complex comprising TSC1, TSC2, and TBC1D7 [97] and functions as a GTPase activating protein (GAP) for the small GTPase Rheb [98, 99]. Activation of AMPK thus inhibiting mTOR in the ischemic heart led to increased autophagy and decreased ischemic injury [86]. Sirtuin3 (SIRT3) is a member of the silent information regulator 2 (Sir2) family of proteins located in mitochondria that influences almost every major aspect of mitochondrial biology [100]. SIRT3 protects against oxygen and glucose deprivation by inducing autophagy through activation of the AMPK thus suppressing mTOR pathway in cortical neurons, indicating that SIRT3 may protect neurons from cerebral ischemia [100]. Recently, it has been shown that conventional protein kinase Cγ (cPKCγ) could alleviate ischemic injury and improve the neurological outcome of mice with ischemic stroke through inhibition of Akt thus inhibiting mTOR pathway to modulate autophagy, providing a potential therapeutic target for ischemic stroke [17].

However, in the clinical setting like atherosclerosis, thromboangitis obliterans and polyarteritis, patients usually experience prolonged periods of chronic ischemia before reperfusion can be reestablished. mTORC1 can be activated in the remote myocardium during chronic myocardial infarction as a consequence of increased load and contributes to ventricular remodeling [101, 102]. Pharmacological mTORC1 inhibition with everolimus reduces cardiac dilation and infarct size and improves cardiac function during chronic myocardial infarction [103]. Zhai and his colleagues revealed that the inhibition of glycogen synthase kinase-3β (GSK-3β) activation was associated with autophagy inhibition and increased ischemic injury through mTORC1 reactivation which was rescued by rapamycin treatment during prolonged myocardial ischemia without reperfusion [104]. Additionally, it is possible that the protective effects in IR injury are dependent on mTORC2 activation, which is required for cardiomyocyte survival during ischemia and limitation of chronic ischemic remodeling [105]. mTORC2 inhibition caused deterioration of cardiac function and remodeling after myocardial infarction. Moreover, PRAS40 overexpression inhibits mTORC1, reduces cardiac remodeling, and improves cardiac function during chronic myocardial infarction [102]. Based on these findings, we propose that mTORC1 inhibition and mTORC2 activation seem to be beneficial during chronic ischemic injury, which highlights the importance of developing new selective mTORC1 inhibitors that do not affect or possibly even increase mTORC2 activity [72].

## The positive aspect of mTOR in IR injury

Reperfusion is mandatory to salvage acutely ischemic tissues from infarction. However, the process of restoring blood ?ow to the ischemic organs or tissues can also contribute to irreversible injury [106, 107], which appears to reflect an oxidant burden established upon reoxygenation of ischemic tissues or organs [84]. IR induces cytosolic and mitochondrial calcium overload, oxidative stress, rapid restoration in intracellular pH, which on a background of relative adenosine triphosphate (ATP) depletion, culminates in the opening of the mitochondrial permeability transition pore (mPTP) and free radical-induced irreversible mitochondrial damage [108]. Advancing age is a strong risk factor for IR injury [109, 110]. The age-related physiological or pathological changes in the cellular components have been shown to increase the vulnerability of IR injury [109-111]. Aging heart is more sensitive to IR injury, and cardiac mitochondrial function has a significant decline in aging, including mitochondrial Ca^2+^ handling decline and mitochondrial ROS generation/oxidative damage [112, 113]. It was demonstrated that mTORC1 is rapidly activated and exerts protective effects during the reperfusion phase [86]. Consistent with the idea that mTORC1 exerts a protective effect during reperfusion damage, cardiac-specific mTOR overexpression reduces chronic cardiac remodeling after IR *in vivo* [72] (Fig. 3). Below we will discuss the different signaling pathways and the protective effects of mTOR regulation in IR injury (Fig. 2B).

### PI3K/Akt and mTOR

The mTORC1/S6K pathway is the major inhibitory signaling that shuts off autophagy and mTORC1 is a key kinase downstream of Akt, thus activation of PI3K inducing the phosphorylation of Akt may result in mTORC1/S6K pathway activation and subsequent autophagy inhibition [18]. Cerebral IR induced brain damage with down-regulation of hepatocyte-growth factor (HGF); astrocytes activation and N-Butylphthalide treatment significantly increased HGF expression, which promoted cMet, a HGF surface receptor, thus stimulating PI3K/Akt/mTORC1 activity and suppressing apoptosis in brain tissues [114]. Stimulating Akt signaling then activating mTOR/S6K pathway may provide potential neuroprotection against IR injury [115]. Moreover, insulin was reported to improve post-ischemic recovery of function through activation of PI3K/Akt thus stimulating mTORC1/S6K1 pathway in isolated working rat hearts independent of glucose uptake during reperfusion [116-118]. Activation of PI3K/Akt signaling thus activating mTOR pathway provided protection for mice against IR-induced acute renal injury [11].

### GSK-3β and mTOR

GSK-3β is known as a multifunctional kinase having more than 40 substrates, playing roles in glycogen metabolism, cell proliferation, growth and death; it has been revealed that suppression of GSK-3β protects against IR injury [119-121]. Activation of mTORC1 by GSK-3β inhibition reduces reperfusion injury by limiting exaggerated activation of autophagy [86, 121, 122]. Reperfusion increasing ROS production causes damage to mitochondrial DNA (mtDNA) thus dysregulating transcriptional factors, as well as disturbs calcium homeostasis and induces lipid peroxidation which subsequently decreases mitochondrial redox potential and leads to the opening of the mPTP and the release of cytochrome c [123, 124], and suppression of GSK-3β exerts an inhibitory effect on mPTP opening induced apoptotic cell death [121, 122]. Moreover, several studies demonstrated that activation of GSK-3β thus inhibiting the pro-survival pathway of PI3k/Akt/mTOR signaling and JAK/STAT3 signaling, finally blunting the cardioprotective effects of IPostC [16, 125-127].

### p38 MAPK and mTOR

p38-MAPK are activated by a wide range of extracellular influences, including radiation, ultraviolet light, heat shock, osmotic stress, proinflammatory cytokines and myocardial ischemia [128-130]. There is considerable evidence that p38 MAPK are activated during myocardial IR injury and growing researches suggest the role of p38 MAPK in the setting of cardioprotection [131]. Recently, Hernández G and his colleagues identified activation of mTOR exerted protection against IR through stimulating p38 MAPK-regulated downstream signaling (i.e., REDD1 and TSC2) in the cardiomyocytes [132].

### HIF-1α and mTOR

Angiogenesis is the physiological process by which new blood vessels sprout from preexisting vessels and it is an important component of protection against IR injury, which has been proved to be mechanically via mTOR-dependent pathway [133, 134]. A key transcriptional regulator of the hypoxic response is HIF, which consists of HIF-1α and HIF-1β subunits. Studies have indicated that HIF-1α is an important oxygen sensor and plays a crucial role in vasculogenesis and angiogenesis [135]. Vascular endothelial growth factors (VEGFs), a particularly key HIF-1α responsive gene, production and signaling are partly dependent on the induction of HIF-1α expression [135, 136]. The inhibition of HIF-1α-mediated VEGF expression can suppress neovascularization [137]. Inhibition of mTOR signaling by rapamycin administration leads to subsequent impaired angiogenesis in aortic endothelial cells [134]. *In vitro* experiments suggest that DEPTOR, an mTOR binding protein that functions to inhibit the mTOR pathway, is crucial for vascular endothelial cell (EC) activation and angiogenic responses. Deptor knockdown led to upregulated expression of CD31 and HIF-1α, and further stimulated the expression of VEGF which promoted angiogenesis [138] (Fig. 3). However, HIF-1α-mediated angiogenic responses following IR at early and late stages are complex and poorly understood. The early stages of IR seem to be associated with an antiangiogenic response, whereas the hypoxia that follows IR at later stages may activate HIF-1α, VEGF, mTOR and may be beneficial by stabilizing the microvasculature and favoring local blood supply [139]. A further finding supported the protection against distant lung injury triggered by renal graft IR injury was likely through activation of mTOR thus enhancing the activity of HIF-1α which attenuated high-mobility group protein-1 (HMGB-1) translocation and nuclear factor-kappa B (NF-κB) activation in A549 cells with oxidative and inflammatory stress [140].

**Table 1:** mTOR is involved in conditioning against IR injury.

Type of organ	Experiment models	Treatments	Mechanisms	Refs.
**Heart**	Isolated perfused rat hearts	IPC	Activation of mTORC1 via stimulating Akt and inhibiting GSK-3β	[156]
	Prolonged ischemia model of Tg-DnGSK-3β or GSK-3β KO mice	Prolonged ischemia without reperfusion	Inhibiting GSK-3β and reactivating mTORC1	[104]
	IR model of Akt KO mice	IPostC	mTOR-dependent GSK-3β inhibition mechanisms	[104]
	IR model of Akt KO mice	GSK-3 inhibitor SB415286 PC	Inhibition of GSK-3β through mTORC1 hyperactivation	[104]
	H/R model of rats	Ghrelin PC	Activation of PI3K/Akt/mTOR/S6K1 signaling pathway	[117, 118]
	Ischemia model of diabetic mice	Rapamycin PC	Inhibition of mTOR via activating the JAK2-STAT3 signaling	[16, 160]
	IR model of mice	Rapamycin PC	p38 MAPK pathway signals through REDD1, Tsc2 to activate mTOR	[132]
	IR model of mice	Rapamycin or DMSO PostC	Selective activation of mTORC2 and ERK with concurrent inhibition of mTORC1 and p38 MAPK	[6]
	IR model of rats	PL PC	Attenuating mTORC1 signaling and inhibiting Beclin-1-dependent pathway	[5, 148]
	IR model of mice	Crocin PC	Activation of AMPK during ischemia while activation of Akt during reperfusion	[79]
	IR model of rats	Epigallocatechin gallate PostC	Inhibiting apoptosis and restoring the autophagic flux via stimulating mTOR	[157]
Brain	IR model of mice	Isoflurane PC	HIF-1α upregulation through stimulating Akt/mTOR/S6K signaling pathway	[115]
	IR model of mice	SMXZF PostC	Inhibition of autophagy provides protection against cerebral IR injury during reperfusion	[83]
	IR model of rats	N-Butylphthalide PostC	Stimulating PI3K/Akt/mTOR activity and suppressing apoptosis	[114]
**Liver**	IR model of rats	Octreotide or octreotide combined with 3-methyladenine PC	Enhancement of autophagy regulated through Akt/mTOR/p70S6K pathway deactivation	[18]
**Kidney**	Stimulated IR model of HUVECs	Rapamycin PC	mTOR inhibits ICAM-1 expression	[47, 143]
	IR model of mice	Aloperine PC	Activation of PI3K/Akt signaling thus activating mTOR and NFκB transcriptional activity	[11]
	Kidney transplantation model of rats	Xenon PostC	Activation of mTOR thus enhancing the activity of HIF-1α	[85]
**Others**	IR model of rats	IPostC	Attenuating autophagy via strengthening mTOR signaling	[13]
	IR model of rats	CAPE PC	Inhibition mTOR reduces the apoptosis on IR damage in rat testis	[15]
	Hindlimb ischemia model of murines	Apelin PostC	Activation of AMPK and inhibition of mTOR during hypoxia while activation of Akt and inhibition of Beclin1during reoxygenation	[14]

### NF-κB and mTOR

NF-κB, a ubiquitous and momentous transcription factor with various stimulants including growth factors and cytokines, which can control the expression of a variety of genes referring to immune responses [141, 142]. mTOR plays a significant role and may lead to innovative therapeutic strategies for treating patients with IR-associated tissue inflammation and organ dysfunction [11, 47, 143]. Inhibition of the NF-κB through activating mTOR could suppress the inflammatory response in myocardial IR injury [144]. Reperfusion of the microvasculature involves the activation of both neutrophils and macrophages which follows a regulated dysfunction after IR injury [145]. Rapamycin potentiates thrombin induced expression of ICAM-1, a cell surface glycoprotein which is typically expressed on endothelial cells and cells of the immune system, via accelerating and stabilizing NF-κB activation in endothelial cells [143], indicating that mTOR negatively regulates ICAM-1 expression in endothelial cells to limit tissue infiltration of leukocytes as well as proinflammatory responses in monocytes and mDCs [47].

## The negative aspect of mTOR in IR injury

Although extensive research suggests that mTOR activation exerts protective effects during IR injury, some studies have shown that mTOR may play a deleterious role in reperfusion injury. Simvastatin reduces IR injury through the inhibition of mTOR and activation of mitophagy [72]. Testicular IR injury is usually induced by torsion/detorsion, and inhibition mTOR reduces the apoptosis on IR damage in rat testis [15, 146, 147]. Additionally, attenuating Akt/mTOR/ p70S6K pathway reduces kidney inflammation and apoptosis after hepatic IR [18]. Suppression of mTORC1 through activation of AMPK results in enhancement of protective autophagy and protects heart and kidney against IR injury [5, 12, 148]. Interestingly, upregulation of SIRT1 inhibits mTOR activity via AMPK activation thus protecting liver grafts from the IR injury associated with orthotopic liver transplantation [149]. It has been reported that mTOR inhibition by rapamycin protects the heart by selective activation of ERK and inhibition of p38 MAPK during reperfusion injury [6]. Moreover, mTORC1 inhibition via restraining the p38 MAPK activation induces protective autophagy cerebral during IR injury [150]. The negative effects of mTOR that is contrary to previous results may be explained by the level of activation of autophagy during reperfusion injury. Autophagy was also induced by ischemia and further enhanced by reperfusion. We can be hypothesized that if the cellular stress is manageable and the activation of autophagy is protective during reperfusion, it would be deleterious to activate mTOR; if the damage is beyond repair and the activation of autophagy is excessive, activation of mTOR would be beneficial.

## The association between mTOR and conditioning phenomena

An increasing number of efforts have attempted to search for proper agents for the treatment of IR injury [16, 125, 126, 148]. There is currently no stronger protection than that by the conditioning phenomena although the effectiveness of conditioning decreases with age [151-153]. Ischemic conditioning means applying brief episodes of nonlethal IR to confer protection against a sustained episode of lethal IR injury, which was originally discovered in 1986 by Murry *et al.* and termed ‘ischemic preconditioning’ [154]. The protective stimulus can be applied before (IPC) or after (ischemic preconditioning) onset of the sustained episode of lethal ischemia, or even at the onset of myocardial reperfusion, which called IPostC [155]. Furthermore, the protective stimulus can be applied by placing a blood-pressure cuff on an upper or lower limb to induce brief episodes of nonlethal ischemia and reperfusion (remote ischemic conditioning, RIC), as well as pharmacological conditioning is applied to clinical with elucidation of the signal-transduction pathways underlying ischemic conditioning [155]. We will describe the role of mTOR in ischemic and pharmacological conditioning hereinafter (Table 1).

A previous study indicated that rapamycin abolished the cardioprotective effects of IPC, indicating that reactive oxygen species (ROS) induced mTORC1 activation via activation of Akt mediating the protection associated with IPC [156]. In addition, IPostC has protective effects on lung IR injury by attenuating autophagy via strengthening mTOR signaling [13]. Rapamycin reduced infarct size *in vivo* IR models when administered before ischemic while rapamycin was not cardioprotective during IR when administered before the reperfusion phase [87, 104], indicating that mTOR activation provides potential protection against IR injury. It has found that isoflurane preconditioning alleviated the IR-induced neurological deficits, infarct volume, brain edema and cell apoptosis via up-regulating HIF-1α expression through stimulating Akt then activating mTOR/S6K signaling pathway [115] (Fig. 3). Liang and his colleagues revealed apelin increases the viability of mesenchymal stem cells via suppressing autophagic cell death through activation of Akt/mTOR during reoxygenation [14]. Crocin, the main effective component of saffron alleviating IR injury via activation of Akt/mTOR during reperfusion [79]. Pretreatment with aloperine provided protection for mice against IR-induced acute renal injury by attenuating inflammatory infiltration, reducing tubular apoptosis along with preserving renal function, which may selectively repress IL-1β and IFN-γ expression via activation of PI3K/Akt signaling thus activating mTOR/NF-κB pathway [11] (Fig. 3). Epigallocatechin gallate postconditioning alleviates myocardial IR injury by inhibiting apoptosis and restoring the autophagic flux via stimulating mTOR [157].

Contrary to popular belief, a recent study documented that myocardial IPostC inhibits cardiac pro-apoptotic signaling and elevates autophagic signaling through mTORC1 inhibition via activation of AMPK and TSC stimulation, resulting in enhancement of protective autophagy and inhibition of excessive autophagy to protect myocardia against IR injury [5, 148]. Octreotide preconditioning enhanced autophagy through Akt/mTOR/p70S6K pathway deactivation and potentially reduced kidney inflammation and apoptosis after hepatic IR [18]. Collectively, accumulating evidence suggests that conditioning provides potential protection against IR injury via regulating mTOR-mediated signaling pathways, and it could be hypothesized that mTOR inhibition during ischemia while mTOR activation during reperfusion through conditioning will be a useful strategy for the treatment of IR injury despite the controversial results in the reperfusion stage.

## Potential future directions

Accumulating evidences derived from experimental models and clinical patients show that mTOR plays an important role in the progression of IR injury [15, 16]. It is still controversial to clearly understand the role of mTOR signaling in reperfusion injury since both protective and toxic effects were observed *in vivo* and *in vitro* [16]. Researchers have found both protective and toxic effects of mTOR signaling when using its inhibitor-rapamycin or transgenic animals [16, 18, 143, 158, 159]. The conflicted outcomes could be explained for five reasons: (i) mTORC1 and mTORC2 have different functions. mTORC1 presents both beneficial and detrimental effects on reperfusion injury while mTORC2 show mostly cardioprotective actions as its cellular survival functions [102, 160]; (ii) mTORC1 phosphorylation site is different. mTORC1 predominately phosphorylated the specific site encompassing 4E-BP1 that are rapamycin resistant as well as phosphorylated S6K1, which is rapamycin sensitive under conditions [16]; (iii) There are degrees of mTOR activation in the regulation of autophagy. Yu *et al.* revealed that mTOR signaling was inhibited during autophagy initially, but reactivated with prolonged autophagy, indicating that the progress was autophagy-dependent and required the degradation of autolysosomal products. In verse, the enhanced mTOR activity attenuated autophagy [161]; (iv) Loss-of-function animal models may have inescapable defects that may influence the results [162, 163]. Although mTOR’s role in reperfusion injury is controversial, a great many of experimental and clinical results proved that activation of mTOR thus inhibiting autophagy during reperfusion reduced IR injury, indicating that mTOR may be theoretically attractive as a therapeutic target.

Autophagy has been implicated in the pathogenesis of IR injury. Perturbation of this evolutionarily conserved intracellular cleansing autophagy mechanism, by targeted modulation through mTOR inhibitors, AMPK modulators, calcium lowering agents, resveratrol, longevinex, sirtuin activators, theproapoptotic gene *Bnip3*, IP3 and lysosome inhibitors, may confer resistance to against IR induced cell death [22]. However, a debate persists as to whether autophagy acts as a protective mechanism or contributes to the injurious effects of IR injury and which of these predominates may depend on the experimental context. The controversy surrounding autophagy in IR injury may stem from several factors including the variability in the experimental models and species, the methodology used to assess autophagy and the severity of ischemia and its duration [22, 164]. In addition, the level of activation of autophagy may dictate the nature of its role [22]. Autophagy is activated as an adaptive response to maintain the survival of cells under stressful conditions while if the cellular stress is not manageable and elicits damage that is beyond repair, the activation of autophagy may drive cells to die [22]. Presently, our knowledge can only hypothesize that autophagy may be protective during ischemia, whereas it may be detrimental during reperfusion. Thus, to precisely define the role of autophagy in IR injury, it is necessary to establish and standardize the experimental models that recapitulate various degrees of ischemic stress.

Ischemic injury is usually accompanied by subsequent reperfusion injury. Reperfusion was reported to significantly alter multiple mitochondrial parameters, including mitochondrial oxygen consumption rates, complex I and complex III activity, H_2_O_2_ production as well as the degree of lipid peroxidation [165]. Mitochondria are the most important effector of conditioning’s protection, where most of the above signaling pathways converge, which are decisive for cellular survival or death, respectively [166]. Aging is associated with an accumulation of pathologic changes leading to a progressive decline in cellular, organ and whole organism function [2]. In addition, aging results in a lower resistance to stress [167]. Mitochondria have been recognized to play a prominent role in aging and age-related diseases, and mitochondrial dysfunction and decline are deeply involved in IR injury of various organs and tissues with aging [2]. Excessive free radical induced by ischemia and subsequent reperfusion directly damages multiple mitochondrial components including respiratory chain, metabolism enzymes, inner mitochondrial membrane depolarization and opening of the mPTP, resulting in mitochondrial malfunction, ATP shortage, and pro-apoptotic factors release. mPTP opening for longer terms results in matrix swelling and the release of cytochrome C from the intermembrane space into the cytosol where it activates proteolytic processes and initiates cellular desintegration on reperfusion [168, 169]. However, transient mPTP opening may serve a physiological function [170] in ROS homeostasis [171] and calcium release [172], and indeed transient mPTP opening is cardioprotective during IPC [173]. Studies found that inhibition of GSK-3β was proposed to integrate all upstream signals and exert an inhibitory effect on mPTP opening [121, 122], the genuine paradox of conditioning—a little injury protects, whereas profound injury is deleterious [166]. Moreover, the damaged mitochondria have impaired mitochondrial dynamics and mitophagy which are crucial for quality control of mitochondrial network [174, 175]. The above mitochondrial changes lead to increased apoptosis and exacerbate IR-induced injury in organs and tissues. Moreover, a previous study revealed that endoplasmic reticulum (ER) stress caused by a buildup of misfolded proteins, implicating in a series of pathophysiological processes, which is able to promote mitochondrial damage under the condition of bacterial infection [176, 177]. However, it has been not validated if mTOR exerts its beneficial roles on IR injury via modulating ER stress-mitochondrial damage axis in multiple organs and tissues.

Clinical trials of mTOR inhibitors in cardiovascular system is limited to drug-eluting stent after coronary heart diseases or artery stenosis. There is a significant benefit of mTOR inhibitors (e.g., rapamycin, everolimus, sirolimus) eluting stents in treating artery stenosis (NCT00350454, NCT00140530, NCT00598676, NCT00332397, NCT01035450, NCT00697372, NCT00231244). Although other mTOR inhibitors (e.g., temsirolimus, deforolimus, tacrolimus, CC-223) have stepped into clinical trials for a variety of uses including cancer treatment (NCT00777959, NCT00483262) and immunosuppression (NCT00619398, NCT0093 1255), it has yet to be tested against a broad spectrum of IR injury (http://clinicaltrials.gov). Interestingly, a recent clinical trial showed that the risk of occurrence of the cardiovascular event is nearly twice as great in renal transplant recipients treated with an mTOR inhibitor, and the incidence of coronary artery diseases during mTOR inhibitor therapy is higher [178]. In the clinical trial of aging (NCT01649960), cellular senescence-associated beta galactosidase activity tended to decrease after rapamycin administration. Furthermore, there are some correlations between some senescence markers and physical performance. Therefore, clinical trials regarding mTOR targeted drugs in the protection of IR warrants further investigation and we anticipate a bounty of additional data on the effects of mTOR in IR injury over the next few years.

## Conclusions

Increasing evidences suggest that mTOR is deeply involved in IR injury of various organs and tissues, including heart, brain, kindey, liver and other organ or tissues [12, 85, 114, 146, 147]. The modulation of mTOR expression appears to be a promising strategy for attenuating IR injury. Autophagy that is under the control of mTOR contributes significantly to the degree of IR injury. However, effects of mTOR and autophagy activation in IR injury or conditioning progression are controversial, we have proposed possible reasons to explain the conflicted outcomes in potential future directions, including the variability in the experimental models and species, the methodology used to assess results, the severity of ischemia and its duration and the level of activation of autophagy [22]. Many of experimental and clinical results revealed that it may play different roles depending on different stages. Activation not excessive autophagy via mTOR inhibition during ischemia while simulation of mTOR thus inhibiting autophagy during reperfusion respectively reduces IR injury. The impressive efficacy and safety of mTOR herald it as a promising agent for the treatment of IR injury. However, the interaction between mTOR and other important cellular processes of IR injury have not been fully explored, which deserves much attention in the future.

## References

[1] Favero G, Franceschetti L, Buffoli B, Moghadasian MH, Reiter RJ, Rodella LF, et al (2017). Melatonin: Protection against age-related cardiac pathology. Ageing Res Rev, 35:336-349. 27884595 10.1016/j.arr.2016.11.007

[2] Wojtovich AP, Nadtochiy SM, Brookes PS, Nehrke K (2012). Ischemic preconditioning: the role of mitochondria and aging. Exp Gerontol, 47:1-7. 22100642 10.1016/j.exger.2011.11.001 PMC3245324

[3] Ma Z, Xin Z, Di W, Yan X, Li X, Reiter RJ, et al (2017). Melatonin and mitochondrial function during ischemia/reperfusion injury. Cell Mol Life Sci. 10.1007/s00018-017-2618-6 PMC11107672 28795196

[4] Chouchani ET, Pell VR, James AM, Work LM, Saeb-Parsy K, Frezza C, et al (2016). A Unifying Mechanism for Mitochondrial Superoxide Production during Ischemia-Reperfusion Injury. Cell Metab, 23:254-263. 26777689 10.1016/j.cmet.2015.12.009

[5] Su HH, Chu YC, Liao JM, Wang YH, Jan MS, Lin CW, et al (2017). Phellinus linteus Mycelium Alleviates Myocardial Ischemia-Reperfusion Injury through Autophagic Regulation. Front Pharmacol, 8:175. 28420993 10.3389/fphar.2017.00175 PMC5378821

[6] Filippone S, Samidurai A, Roh S, Cain C, He J, Salloum F, et al (2017). Reperfusion Therapy with Rapamycin Attenuates Myocardial Infarction through Activation of AKT and ERK. Oxid Med Cell Longev, 2017:4619720. 28373901 10.1155/2017/4619720 PMC5360974

[7] Zheng Y, Bu J, Yu L, Chen J, Liu H (2017). Nobiletin improves propofol-induced neuroprotection via regulating Akt/mTOR and TLR 4/NF-kappaB signaling in ischemic brain injury in rats. Biomed Pharmacother, 91:494-503. 28478273 10.1016/j.biopha.2017.04.048

[8] Wu ZQ, Cui SY, Zhu L, Zou ZQ (2016). Study on the Mechanism of mTOR-Mediated Autophagy during Electroacupuncture Pretreatment against Cerebral Ischemic Injury. 2016:9121597. 10.1155/2016/9121597 PMC4980529 27547233

[9] Yang H, Li L, Zhou K, Wang Y, Guan T, Chai C, et al (2016). Shengmai injection attenuates the cerebral ischemia/reperfusion induced autophagy via modulation of the AMPK, mTOR and JNK pathways. Pharm Biol, 54:2288-2297. 26983890 10.3109/13880209.2016.1155625

[10] Zhu J, Lu T, Yue S, Shen X, Gao F, Busuttil RW, et al (2015). Rapamycin protection of livers from ischemia and reperfusion injury is dependent on both autophagy induction and mammalian target of rapamycin complex 2-Akt activation. Transplantation, 99:48-55. 25340604 10.1097/TP.0000000000000476 PMC4272660

[11] Hu S, Zhang Y, Zhang M, Guo Y, Yang P, Zhang S, et al (2015). Aloperine protects mice against ischemia reperfusion (IR)-induced renal injury by regulating PI3K/AKT/mTOR signaling and AP-1 activity. Mol Med. 10.2119/molmed.2015.00056 PMC4818254 26552059

[12] Pu T, Liao XH, Sun H, Guo H, Jiang X, Peng JB, et al (2017). Augmenter of liver regeneration regulates autophagy in renal ischemia-reperfusion injury via the AMPK/mTOR pathway. Apoptosis, 22:955-969. 28466106 10.1007/s10495-017-1370-6

[13] Duan M, Fu Y, Lan J, Wu Y, Xu S, Bai Y (2014). Effects of postconditioning on autophagy of lung ischemic reperfusion injury in rats. Zhonghua Yi Xue Za Zhi, 94:1577-1580. 25146749

[14] Liang D, Han D, Fan W, Zhang R, Qiao H, Fan M, et al (2016). Therapeutic efficacy of apelin on transplanted mesenchymal stem cells in hindlimb ischemic mice via regulation of autophagy. Sci Rep, 6:21914. 26902855 10.1038/srep21914 PMC4763210

[15] Dilber Y, Inan S, Ercan GA, Sencan A (2016). The role of CAPE in PI3K/AKT/mTOR activation and oxidative stress on testis torsion. Acta Histochem, 118:31-37. 26651953 10.1016/j.acthis.2015.11.004

[16] Zhao D, Yang J (2017). Insights for Oxidative Stress and mTOR Signaling in Myocardial Ischemia/Reperfusion Injury under Diabetes. 2017:6437467. 10.1155/2017/6437467 PMC5337354 28298952

[17] Wei H, Li Y, Han S, Liu S, Zhang N, Zhao L, et al (2016). cPKCgamma-Modulated Autophagy in Neurons Alleviates Ischemic Injury in Brain of Mice with Ischemic Stroke Through Akt-mTOR Pathway. Transl Stroke Res, 7:497-511. 27510769 10.1007/s12975-016-0484-4

[18] Sun H, Zou S, Candiotti KA, Peng Y, Zhang Q, Xiao W, et al (2017). Octreotide Attenuates Acute Kidney Injury after Hepatic Ischemia and Reperfusion by Enhancing Autophagy. Sci Rep, 7:42701. 28205545 10.1038/srep42701 PMC5311976

[19] Laplante M, Sabatini DM (2012). mTOR signaling in growth control and disease. Cell, 149:274-293. 22500797 10.1016/j.cell.2012.03.017 PMC3331679

[20] Saxton RA, Sabatini DM (2017). mTOR Signaling in Growth, Metabolism, and Disease. Cell, 169:361-371. 10.1016/j.cell.2017.03.035 28388417

[21] Johnson SC, Rabinovitch PS, Kaeberlein M (2013). mTOR is a key modulator of ageing and age-related disease. Nature, 493:338-345. 23325216 10.1038/nature11861 PMC3687363

[22] Chen-Scarabelli C, Agrawal PR, Saravolatz L, Abuniat C, Scarabelli G, Stephanou A, et al (2014). The role and modulation of autophagy in experimental models of myocardial ischemia-reperfusion injury. J Geriatr Cardiol, 11:338-348. 25593583 10.11909/j.issn.1671-5411.2014.01.009 PMC4294150

[23] Benjamin D, Colombi M, Moroni C, Hall MN (2011). Rapamycin passes the torch: a new generation of mTOR inhibitors. Nat Rev Drug Discov, 10:868-880. 22037041 10.1038/nrd3531

[24] Cafferkey R, Young PR, McLaughlin MM, Bergsma DJ, Koltin Y, Sathe GM, et al (1993). Dominant missense mutations in a novel yeast protein related to mammalian phosphatidylinositol 3-kinase and VPS34 abrogate rapamycin cytotoxicity. Mol Cell Biol, 13:6012-6023. 8413204 10.1128/mcb.13.10.6012-6023.1993 PMC364661

[25] Kunz J, Henriquez R, Schneider U, Deuter-Reinhard M, Movva NR, Hall MN (1993). Target of rapamycin in yeast, TOR2, is an essential phosphatidylinositol kinase homolog required for G1 progression. Cell, 73:585-596. 8387896 10.1016/0092-8674(93)90144-f

[26] Loewith R, Jacinto E, Wullschleger S, Lorberg A, Crespo JL, Bonenfant D, et al (2002). Two TOR complexes, only one of which is rapamycin sensitive, have distinct roles in cell growth control. Mol Cell, 10:457-468. 12408816 10.1016/s1097-2765(02)00636-6

[27] Kim DH, Sarbassov DD, Ali SM, King JE, Latek RR, Erdjument-Bromage H, et al (2002). mTOR interacts with raptor to form a nutrient-sensitive complex that signals to the cell growth machinery. Cell, 110:163-175. 12150925 10.1016/s0092-8674(02)00808-5

[28] Kim DH, Sarbassov DD, Ali SM, Latek RR, Guntur KV, Erdjument-Bromage H, et al (2003). GbetaL, a positive regulator of the rapamycin-sensitive pathway required for the nutrient-sensitive interaction between raptor and mTOR. Mol Cell, 11:895-904. 12718876 10.1016/s1097-2765(03)00114-x

[29] Hara K, Maruki Y, Long X, Yoshino K, Oshiro N, Hidayat S, et al (2002). Raptor, a binding partner of target of rapamycin (TOR), mediates TOR action. Cell, 110:177-189. 12150926 10.1016/s0092-8674(02)00833-4

[30] Sancak Y, Thoreen CC, Peterson TR, Lindquist RA, Kang SA, Spooner E, et al (2007). PRAS40 is an insulin-regulated inhibitor of the mTORC1 protein kinase. Mol Cell, 25:903-915. 17386266 10.1016/j.molcel.2007.03.003

[31] Vander Haar E, Lee SI, Bandhakavi S, Griffin TJ, Kim DH (2007). Insulin signalling to mTOR mediated by the Akt/PKB substrate PRAS40. Nat Cell Biol, 9:316-323. 17277771 10.1038/ncb1547

[32] Peterson TR, Laplante M, Thoreen CC, Sancak Y, Kang SA, Kuehl WM, et al (2009). DEPTOR is an mTOR inhibitor frequently overexpressed in multiple myeloma cells and required for their survival. Cell, 137:873-886. 19446321 10.1016/j.cell.2009.03.046 PMC2758791

[33] Kaizuka T, Hara T, Oshiro N, Kikkawa U, Yonezawa K, Takehana K, et al (2010). Tti1 and Tel2 are critical factors in mammalian target of rapamycin complex assembly. J Biol Chem, 285:20109-20116. 20427287 10.1074/jbc.M110.121699 PMC2888423

[34] Nojima H, Tokunaga C, Eguchi S, Oshiro N, Hidayat S, Yoshino K, et al (2003). The mammalian target of rapamycin (mTOR) partner, raptor, binds the mTOR substrates p70 S6 kinase and 4E-BP1 through their TOR signaling (TOS) motif. J Biol Chem, 278:15461-15464. 12604610 10.1074/jbc.C200665200

[35] Schalm SS, Fingar DC, Sabatini DM, Blenis J (2003). TOS motif-mediated raptor binding regulates 4E-BP1 multisite phosphorylation and function. Curr Biol, 13:797-806. 12747827 10.1016/s0960-9822(03)00329-4

[36] Sancak Y, Peterson TR, Shaul YD, Lindquist RA, Thoreen CC, Bar-Peled L, et al (2008). The Rag GTPases bind raptor and mediate amino acid signaling to mTORC1. Science, 320:1496-1501. 18497260 10.1126/science.1157535 PMC2475333

[37] Yang H, Rudge DG, Koos JD, Vaidialingam B, Yang HJ, Pavletich NP (2013). mTOR kinase structure, mechanism and regulation. Nature, 497:217-223. 23636326 10.1038/nature12122 PMC4512754

[38] Guertin DA, Stevens DM, Thoreen CC, Burds AA, Kalaany NY, Moffat J, et al (2006). Ablation in mice of the mTORC components raptor, rictor, or mLST8 reveals that mTORC2 is required for signaling to Akt-FOXO and PKCalpha, but not S6K1. Dev Cell, 11:859-871. 17141160 10.1016/j.devcel.2006.10.007

[39] Pearce LR, Huang X, Boudeau J, Pawlowski R, Wullschleger S, Deak M, et al (2007). Identification of Protor as a novel Rictor-binding component of mTOR complex-2. Biochem J, 405:513-522. 17461779 10.1042/BJ20070540 PMC2267312

[40] Thedieck K, Polak P, Kim ML, Molle KD, Cohen A, Jeno P, et al (2007). PRAS40 and PRR5-like protein are new mTOR interactors that regulate apoptosis. PLoS One, 2:e1217. 18030348 10.1371/journal.pone.0001217 PMC2075366

[41] Woo SY, Kim DH, Jun CB, Kim YM, Haar EV, Lee SI, et al (2007). PRR5, a novel component of mTOR complex 2, regulates platelet-derived growth factor receptor beta expression and signaling. J Biol Chem, 282:25604-25612. 17599906 10.1074/jbc.M704343200

[42] Frias MA, Thoreen CC, Jaffe JD, Schroder W, Sculley T, Carr SA, et al (2006). mSin1 is necessary for Akt/PKB phosphorylation, and its isoforms define three distinct mTORC2s. Curr Biol, 16:1865-1870. 16919458 10.1016/j.cub.2006.08.001

[43] Jacinto E, Facchinetti V, Liu D, Soto N, Wei S, Jung SY, et al (2006). SIN1/MIP1 maintains rictor-mTOR complex integrity and regulates Akt phosphorylation and substrate specificity. Cell, 127:125-137. 16962653 10.1016/j.cell.2006.08.033

[44] Yang Q, Inoki K, Ikenoue T, Guan KL (2006). Identification of Sin1 as an essential TORC2 component required for complex formation and kinase activity. Genes Dev, 20:2820-2832. 17043309 10.1101/gad.1461206 PMC1619946

[45] Jacinto E, Loewith R, Schmidt A, Lin S, Ruegg MA, Hall A, et al (2004). Mammalian TOR complex 2 controls the actin cytoskeleton and is rapamycin insensitive. Nat Cell Biol, 6:1122-1128. 15467718 10.1038/ncb1183

[46] Sarbassov DD, Ali SM, Kim DH, Guertin DA, Latek RR, Erdjument-Bromage H, et al (2004). Rictor, a novel binding partner of mTOR, defines a rapamycin-insensitive and raptor-independent pathway that regulates the cytoskeleton. Curr Biol, 14:1296-1302. 15268862 10.1016/j.cub.2004.06.054

[47] Saemann MD, Haidinger M, Hecking M, Horl WH, Weichhart T (2009). The multifunctional role of mTOR in innate immunity: implications for transplant immunity. Am J Transplant, 9:2655-2661. 19788500 10.1111/j.1600-6143.2009.02832.x

[48] Lamming DW, Ye L, Katajisto P, Goncalves MD, Saitoh M, Stevens DM, et al (2012). Rapamycin-induced insulin resistance is mediated by mTORC2 loss and uncoupled from longevity. Science, 335:1638-1643. 22461615 10.1126/science.1215135 PMC3324089

[49] Sarbassov DD, Ali SM, Sengupta S, Sheen JH, Hsu PP, Bagley AF, et al (2006). Prolonged rapamycin treatment inhibits mTORC2 assembly and Akt/PKB. Mol Cell, 22:159-168. 16603397 10.1016/j.molcel.2006.03.029

[50] Cao W, Manicassamy S, Tang H, Kasturi SP, Pirani A, Murthy N, et al (2008). Toll-like receptor-mediated induction of type I interferon in plasmacytoid dendritic cells requires the rapamycin-sensitive PI(3)K-mTOR-p70S6K pathway. Nat Immunol, 9:1157-1164. 18758466 10.1038/ni.1645 PMC3732485

[51] Schmitz F, Heit A, Dreher S, Eisenacher K, Mages J, Haas T, et al (2008). Mammalian target of rapamycin (mTOR) orchestrates the defense program of innate immune cells. Eur J Immunol, 38:2981-2992. 18924132 10.1002/eji.200838761

[52] Jacinto E, Lorberg A (2008). TOR regulation of AGC kinases in yeast and mammals. Biochem J, 410:19-37. 18215152 10.1042/BJ20071518

[53] Gingras AC, Raught B, Sonenberg N (2001). Regulation of translation initiation by FRAP/mTOR. Genes Dev, 15:807-826. 11297505 10.1101/gad.887201

[54] Inoki K, Kim J, Guan KL (2012). AMPK and mTOR in cellular energy homeostasis and drug targets. Annu Rev Pharmacol Toxicol, 52:381-400. 22017684 10.1146/annurev-pharmtox-010611-134537

[55] Hellsten Y, Richter EA, Kiens B, Bangsbo J (1999). AMP deamination and purine exchange in human skeletal muscle during and after intense exercise. J Physiol, 520 Pt 3: 909-920. 10.1111/j.1469-7793.1999.00909.x PMC2269626 10545153

[56] McBride A, Hardie DG (2009). AMP-activated protein kinase--a sensor of glycogen as well as AMP and ATP? Acta Physiol (Oxf), 196:99-113. 19245651 10.1111/j.1748-1716.2009.01975.x

[57] Dibble CC, Asara JM, Manning BD (2009). Characterization of Rictor phosphorylation sites reveals direct regulation of mTOR complex 2 by S6K1. Mol Cell Biol, 29:5657-5670. 19720745 10.1128/MCB.00735-09 PMC2772744

[58] Oakhill JS, Scott JW, Kemp BE (2009). Structure and function of AMP-activated protein kinase. Acta Physiol (Oxf), 196:3-14. 19245650 10.1111/j.1748-1716.2009.01977.x

[59] Xiao B, Sanders MJ, Underwood E, Heath R, Mayer FV, Carmena D, et al (2011). Structure of mammalian AMPK and its regulation by ADP. Nature, 472:230-233. 21399626 10.1038/nature09932 PMC3078618

[60] Das S, Aiba T, Rosenberg M, Hessler K, Xiao C, Quintero PA, et al (2012). Pathological role of serum- and glucocorticoid-regulated kinase 1 in adverse ventricular remodeling. Circulation, 126:2208-2219. 23019294 10.1161/CIRCULATIONAHA.112.115592 PMC3484211

[61] Xiao B, Heath R, Saiu P, Leiper FC, Leone P, Jing C, et al (2007). Structural basis for AMP binding to mammalian AMP-activated protein kinase. Nature, 449:496-500. 17851531 10.1038/nature06161

[62] Kennedy BK, Kaeberlein M (2009). Hot topics in aging research: protein translation, 2009. Aging Cell, 8:617-623. 19747234 10.1111/j.1474-9726.2009.00522.x PMC3673879

[63] Wieser D, Papatheodorou I, Ziehm M, Thornton JM (2011). Computational biology for ageing. Philos Trans R Soc Lond B Biol Sci, 366:51-63. 21115530 10.1098/rstb.2010.0286 PMC3001313

[64] AbouRjaili G, Shtaynberg N, Wetz R, Costantino T, Abela GS (2010). Current concepts in triglyceride metabolism, pathophysiology, and treatment. Metabolism, 59:1210-1220. 20060141 10.1016/j.metabol.2009.11.014

[65] Kennedy BK, Pennypacker JK (2016). Mammalian Target of Rapamycin: A Target for (Lung) Diseases and Aging. Ann Am Thorac Soc, 13:S398-s401. 28005429 10.1513/AnnalsATS.201609-680AW PMC5291471

[66] Newgard CB, Sharpless NE (2013). Coming of age: molecular drivers of aging and therapeutic opportunities. J Clin Invest, 123:946-950. 23454756 10.1172/JCI68833 PMC3582156

[67] Avruch J, Hara K, Lin Y, Liu M, Long X, Ortiz-Vega S, et al (2006). Insulin and amino-acid regulation of mTOR signaling and kinase activity through the Rheb GTPase. Oncogene, 25:6361-6372. 17041622 10.1038/sj.onc.1209882

[68] Kennedy BK, Lamming DW (2016). The Mechanistic Target of Rapamycin: The Grand ConducTOR of Metabolism and Aging. Cell Metab, 23:990-1003. 27304501 10.1016/j.cmet.2016.05.009 PMC4910876

[69] Wilkinson JE, Burmeister L, Brooks SV, Chan CC, Friedline S, Harrison DE, et al (2012). Rapamycin slows aging in mice. Aging Cell, 11:675-682. 22587563 10.1111/j.1474-9726.2012.00832.x PMC3434687

[70] Sokollik C, Ang M, Jones N (2011). Autophagy: a primer for the gastroenterologist/hepatologist. Can J Gastroenterol, 25:667-674. 22175057 10.1155/2011/581264 PMC3266158

[71] Cuervo AM (2008). Autophagy and aging: keeping that old broom working. Trends Genet, 24:604-612. 18992957 10.1016/j.tig.2008.10.002 PMC2745226

[72] Sciarretta S, Volpe M, Sadoshima J (2014). Mammalian target of rapamycin signaling in cardiac physiology and disease. Circ Res, 114:549-564. 24481845 10.1161/CIRCRESAHA.114.302022 PMC3995130

[73] Rosenbluth J, Mays D, Pino M, Tang L, Pietenpol J (2008). A gene signature-based approach identifies mTOR as a regulator of p73. Mol. Cell. Biol., 28:5951-5964. 18678646 10.1128/MCB.00305-08 PMC2547001

[74] Martina J, Chen Y, Gucek M, Puertollano R (2012). MTORC1 functions as a transcriptional regulator of autophagy by preventing nuclear transport of TFEB. Autophagy, 8:903-914. 22576015 10.4161/auto.19653 PMC3427256

[75] Settembre C, Di Malta C, Polito V, Garcia Arencibia M, Vetrini F, Erdin S, et al (2011). TFEB links autophagy to lysosomal biogenesis. Science, 332:1429-1433. 21617040 10.1126/science.1204592 PMC3638014

[76] Egan DF, Shackelford DB, Mihaylova MM, Gelino S, Kohnz RA, Mair W, et al (2011). Phosphorylation of ULK1 (hATG1) by AMP-activated protein kinase connects energy sensing to mitophagy. Science, 331:456-461. 21205641 10.1126/science.1196371 PMC3030664

[77] Levine B, Kroemer G (2008). Autophagy in the pathogenesis of disease. Cell, 132:27-42. 18191218 10.1016/j.cell.2007.12.018 PMC2696814

[78] Mizushima N, Levine B, Cuervo AM, Klionsky DJ (2008). Autophagy fights disease through cellular self-digestion. Nature, 451:1069-1075. 18305538 10.1038/nature06639 PMC2670399

[79] Zeng C, Li H, Fan Z, Zhong L, Guo Z, Guo Y, et al (2016). Crocin-Elicited Autophagy Rescues Myocardial Ischemia/Reperfusion Injury via Paradoxical Mechanisms. Am J Chin Med, 44:515-530. 27109157 10.1142/S0192415X16500282

[80] Yitzhaki S, Huang C, Liu W, Lee Y, Gustafsson AB, Mentzer RM Jr., et al (2009). Autophagy is required for preconditioning by the adenosine A1 receptor-selective agonist CCPA. Basic Res Cardiol, 104:157-167. 19242639 10.1007/s00395-009-0006-6 PMC3682823

[81] Wei C, Li H, Han L, Zhang L, Yang X (2013). Activation of autophagy in ischemic postconditioning contributes to cardioprotective effects against ischemia/reperfusion injury in rat hearts. J Cardiovasc Pharmacol, 61:416-422. 23364609 10.1097/FJC.0b013e318287d501

[82] Valentim L, Laurence KM, Townsend PA, Carroll CJ, Soond S, Scarabelli TM, et al (2006). Urocortin inhibits Beclin1-mediated autophagic cell death in cardiac myocytes exposed to ischaemia/reperfusion injury. J Mol Cell Cardiol, 40:846-852. 16697404 10.1016/j.yjmcc.2006.03.428

[83] Guo Z, Cao G, Yang H, Zhou H, Li L, Cao Z, et al (2014). A combination of four active compounds alleviates cerebral ischemia-reperfusion injury in correlation with inhibition of autophagy and modulation of AMPK/mTOR and JNK pathways. J Neurosci Res, 92:1295-1306. 24801159 10.1002/jnr.23400

[84] Yao H, Han X, Han X (2014). The cardioprotection of the insulin-mediated PI3K/Akt/mTOR signaling pathway. Am J Cardiovasc Drugs, 14:433-442. 25160498 10.1007/s40256-014-0089-9

[85] Fan W, Han D, Sun Z, Ma S, Gao L, Chen J, et al (2017). Endothelial deletion of mTORC1 protects against hindlimb ischemia in diabetic mice via activation of autophagy, attenuation of oxidative stress and alleviation of inflammation. Free Radic Biol Med, 108:725-740. 28473248 10.1016/j.freeradbiomed.2017.05.001

[86] Matsui Y, Takagi H, Qu X, Abdellatif M, Sakoda H, Asano T, et al (2007). Distinct roles of autophagy in the heart during ischemia and reperfusion: roles of AMP-activated protein kinase and Beclin 1 in mediating autophagy. Circ. Res., 100:914-922. 17332429 10.1161/01.RES.0000261924.76669.36

[87] Das A, Salloum F, Durrant D, Ockaili R, Kukreja R (2012). Rapamycin protects against myocardial ischemia-reperfusion injury through JAK2-STAT3 signaling pathway. J Mol Cell Cardiol, 53:858-869. 22999860 10.1016/j.yjmcc.2012.09.007 PMC3496042

[88] Long X, Lin Y, Ortiz-Vega S, Yonezawa K, Avruch J (2005). Rheb binds and regulates the mTOR kinase. Curr Biol, 15:702-713. 15854902 10.1016/j.cub.2005.02.053

[89] Sciarretta S, Zhai P, Shao D, Maejima Y, Robbins J, Volpe M, et al (2012). Rheb is a critical regulator of autophagy during myocardial ischemia: pathophysiological implications in obesity and metabolic syndrome. Circulation, 125:1134-1146. 22294621 10.1161/CIRCULATIONAHA.111.078212 PMC3337789

[90] Wu X, Cao Y, Nie J, Liu H, Lu S, Hu X, et al (2013). Genetic and pharmacological inhibition of Rheb1-mTORC1 signaling exerts cardioprotection against adverse cardiac remodeling in mice. Am J Pathol, 182:2005-2014. 23567640 10.1016/j.ajpath.2013.02.012

[91] Zong H, Li X, Lin H, Hou C, Ma F (2017). Lipoxin A4 pretreatment mitigates skeletal muscle ischemia-reperfusion injury in rats. Am J Transl Res, 9:1139-1150. 28386340 PMC5376005

[92] Hardie DG (2011). AMP-activated protein kinase: an energy sensor that regulates all aspects of cell function. Genes Dev, 25:1895-1908. 21937710 10.1101/gad.17420111 PMC3185962

[93] Kim TW, Kim YJ, Kim HT, Park SR, Lee MY, Park YD, et al (2016). NQO1 Deficiency Leads Enhanced Autophagy in Cisplatin-Induced Acute Kidney Injury Through the AMPK/TSC2/mTOR Signaling Pathway. Antioxid Redox Signal, 24:867-883. 26935540 10.1089/ars.2015.6386

[94] Duan P, Hu C, Quan C, Yu T, Zhou W, Yuan M, et al (2016). 4-Nonylphenol induces apoptosis, autophagy and necrosis in Sertoli cells: Involvement of ROS-mediated AMPK/AKT-mTOR and JNK pathways. Toxicology, 341-343:28-40. 26804764 10.1016/j.tox.2016.01.004

[95] Gwinn DM, Shackelford DB, Egan DF, Mihaylova MM, Mery A, Vasquez DS, et al (2008). AMPK phosphorylation of raptor mediates a metabolic checkpoint. Mol Cell, 30:214-226. 18439900 10.1016/j.molcel.2008.03.003 PMC2674027

[96] Shaw RJ, Bardeesy N, Manning BD, Lopez L, Kosmatka M, DePinho RA, et al (2004). The LKB1 tumor suppressor negatively regulates mTOR signaling. Cancer Cell, 6:91-99. 15261145 10.1016/j.ccr.2004.06.007

[97] Dibble CC, Elis W, Menon S, Qin W, Klekota J, Asara JM, et al (2012). TBC1D7 is a third subunit of the TSC1-TSC2 complex upstream of mTORC1. Mol Cell, 47:535-546. 22795129 10.1016/j.molcel.2012.06.009 PMC3693578

[98] Inoki K, Li Y, Xu T, Guan KL (2003). Rheb GTPase is a direct target of TSC2 GAP activity and regulates mTOR signaling. Genes Dev, 17:1829-1834. 12869586 10.1101/gad.1110003 PMC196227

[99] Tee AR, Manning BD, Roux PP, Cantley LC, Blenis J (2003). Tuberous sclerosis complex gene products, Tuberin and Hamartin, control mTOR signaling by acting as a GTPase-activating protein complex toward Rheb. Curr Biol, 13:1259-1268. 12906785 10.1016/s0960-9822(03)00506-2

[100] Dai SH, Chen T, Li X, Yue KY, Luo P, Yang LK, et al (2017). Sirt3 confers protection against neuronal ischemia by inducing autophagy: Involvement of the AMPK-mTOR pathway. Free Radic Biol Med, 108:345-353. 28396174 10.1016/j.freeradbiomed.2017.04.005

[101] Buss SJ, Muenz S, Riffel JH, Malekar P, Hagenmueller M, Weiss CS, et al (2009). Beneficial effects of Mammalian target of rapamycin inhibition on left ventricular remodeling after myocardial infarction. J Am Coll Cardiol, 54:2435-2446. 20082935 10.1016/j.jacc.2009.08.031

[102] Volkers M, Konstandin MH, Doroudgar S, Toko H, Quijada P, Din S, et al (2013). Mechanistic target of rapamycin complex 2 protects the heart from ischemic damage. Circulation, 128:2132-2144. 24008870 10.1161/CIRCULATIONAHA.113.003638 PMC4131547

[103] Buss S, Muenz S, Riffel J, Malekar P, Hagenmueller M, Weiss C, et al (2009). Beneficial effects of Mammalian target of rapamycin inhibition on left ventricular remodeling after myocardial infarction. J Am Coll Cardiol, 54:2435-2446. 20082935 10.1016/j.jacc.2009.08.031

[104] Zhai P, Sciarretta S, Galeotti J, Volpe M, Sadoshima J (2011). Differential roles of GSK-3beta during myocardial ischemia and ischemia/reperfusion. Circ Res, 109:502-511. 21737790 10.1161/CIRCRESAHA.111.249532 PMC3158807

[105] Völkers M, Konstandin M, Doroudgar S, Toko H, Quijada P, Din S, et al (2013). Mechanistic target of rapamycin complex 2 protects the heart from ischemic damage. Circulation, 128:2132-2144. 24008870 10.1161/CIRCULATIONAHA.113.003638 PMC4131547

[106] Heusch G (2004). Postconditioning: old wine in a new bottle? J Am Coll Cardiol, 44:1111-1112. 15337226 10.1016/j.jacc.2004.06.013

[107] Bainey KR, Armstrong PW (2014). Clinical perspectives on reperfusion injury in acute myocardial infarction. Am Heart J, 167:637-645. 24766972 10.1016/j.ahj.2014.01.015

[108] Bulluck H, Hausenloy DJ (2015). Ischaemic conditioning: are we there yet? Heart, 101:1067-1077. 25887783 10.1136/heartjnl-2014-306531

[109] Cai W, Zhang K, Li P, Zhu L, Xu J, Yang B, et al (2017). Dysfunction of the neurovascular unit in ischemic stroke and neurodegenerative diseases: An aging effect. Ageing Res Rev, 34:77-87. 27697546 10.1016/j.arr.2016.09.006 PMC5384332

[110] Christoffersen M, Tybjaerg-Hansen A (2016). Visible aging signs as risk markers for ischemic heart disease: Epidemiology, pathogenesis and clinical implications. Ageing Res Rev, 25:24-41. 26590331 10.1016/j.arr.2015.11.002

[111] Nagata K, Yamazaki T, Takano D, Maeda T, Fujimaki Y, Nakase T, et al (2016). Cerebral circulation in aging. Ageing Res Rev, 30:49-60. 27484894 10.1016/j.arr.2016.06.001

[112] Lesnefsky EJ, He D, Moghaddas S, Hoppel CL (2006). Reversal of mitochondrial defects before ischemia protects the aged heart. Faseb j, 20:1543-1545. 16793872 10.1096/fj.05-4535fje

[113] Lesnefsky EJ, Moghaddas S, Tandler B, Kerner J, Hoppel CL (2001). Mitochondrial dysfunction in cardiac disease: ischemia--reperfusion, aging, and heart failure. J Mol Cell Cardiol, 33:1065-1089. 11444914 10.1006/jmcc.2001.1378

[114] Zhang P, Guo ZF, Xu YM, Li YS, Song JG (2016). N-Butylphthalide (NBP) ameliorated cerebral ischemia reperfusion-induced brain injury via HGF-regulated TLR4/NF-kappaB signaling pathway. Biomed Pharmacother, 83:658-666. 27468961 10.1016/j.biopha.2016.07.040

[115] Yan W, Chen Z, Chen J, Chen H (2016). Isoflurane preconditioning protects rat brain from ischemia reperfusion injury via up-regulating the HIF-1alpha expression through Akt/mTOR/s6K activation. Cell Mol Biol (Noisy-le-grand), 62:38-44. 26950449

[116] Iliadis F, Kadoglou N, Didangelos T (2011). Insulin and the heart. Diabetes Res Clin Pract, 93 Suppl 1:S86-91. 21864757 10.1016/S0168-8227(11)70019-5

[117] Wang L, Lu Y, Liu X, Wang X (2017). Ghrelin protected neonatal rat cardiomyocyte against hypoxia/reoxygenation injury by inhibiting apoptosis through Akt-mTOR signal. Mol Biol Rep, 44:219-226. 28281036 10.1007/s11033-017-4098-z

[118] Park BM, Cha SA, Lee SH, Kim SH (2016). Angiotensin IV protects cardiac reperfusion injury by inhibiting apoptosis and inflammation via AT4R in rats. Peptides, 79:66-74. 27038740 10.1016/j.peptides.2016.03.017

[119] Jope RS, Yuskaitis CJ, Beurel E (2007). Glycogen synthase kinase-3 (GSK3): inflammation, diseases, and therapeutics. Neurochem Res, 32:577-595. 16944320 10.1007/s11064-006-9128-5 PMC1970866

[120] Jope RS, Johnson GV (2004). The glamour and gloom of glycogen synthase kinase-3. Trends Biochem Sci, 29:95-102. 15102436 10.1016/j.tibs.2003.12.004

[121] Juhaszova M, Zorov DB, Yaniv Y, Nuss HB, Wang S, Sollott SJ (2009). Role of glycogen synthase kinase-3beta in cardioprotection. Circ Res, 104:1240-1252. 19498210 10.1161/CIRCRESAHA.109.197996 PMC2726042

[122] Juhaszova M, Zorov DB, Kim SH, Pepe S, Fu Q, Fishbein KW, et al (2004). Glycogen synthase kinase-3beta mediates convergence of protection signaling to inhibit the mitochondrial permeability transition pore. J Clin Invest, 113:1535-1549. 15173880 10.1172/JCI19906 PMC419483

[123] Govender J, Loos B, Marais E, Engelbrecht AM (2014). Mitochondrial catastrophe during doxorubicin-induced cardiotoxicity: a review of the protective role of melatonin. J Pineal Res, 57:367-380. 25230823 10.1111/jpi.12176

[124] Zhang YW, Shi J, Li YJ, Wei L (2009). Cardiomyocyte death in doxorubicin-induced cardiotoxicity. Arch Immunol Ther Exp (Warsz), 57:435-445. 19866340 10.1007/s00005-009-0051-8 PMC2809808

[125] Lecour S (2009). Activation of the protective Survivor Activating Factor Enhancement (SAFE) pathway against reperfusion injury: Does it go beyond the RISK pathway? J Mol Cell Cardiol, 47:32-40. 19344728 10.1016/j.yjmcc.2009.03.019

[126] Zhuo C, Wang Y, Wang X, Wang Y, Chen Y (2011). Cardioprotection by ischemic postconditioning is abolished in depressed rats: role of Akt and signal transducer and activator of transcription-3. Mol Cell Biochem, 346:39-47. 20830508 10.1007/s11010-010-0589-0

[127] Downey J, Krieg T, Cohen M (2008). Mapping preconditioning’s signaling pathways: an engineering approach. Ann. N. Y. Acad. Sci., 1123:187-196. 10.1196/annals.1420.022 18375591

[128] Sugden PH, Clerk A (1998). "Stress-responsive" mitogen-activated protein kinases (c-Jun N-terminal kinases and p38 mitogen-activated protein kinases) in the myocardium. Circ Res, 83:345-352. 9721691 10.1161/01.res.83.4.345

[129] Ping P, Murphy E (2000). Role of p38 mitogen-activated protein kinases in preconditioning: a detrimental factor or a protective kinase? Circ Res, 86:921-922. 10807861 10.1161/01.res.86.9.921

[130] Luss H, Neumann J, Schmitz W, Schulz R, Heusch G (2000). The stress-responsive MAP kinase p38 is activated by low-flow ischemia in the in situ porcine heart. J Mol Cell Cardiol, 32:1787-1794. 11013123 10.1006/jmcc.2000.1213

[131] Steenbergen C (2002). The role of p38 mitogen-activated protein kinase in myocardial ischemia/reperfusion injury; relationship to ischemic preconditioning. Basic Res Cardiol, 97:276-285. 12111037 10.1007/s00395-002-0364-9

[132] Hernández G, Lal H, Fidalgo M, Guerrero A, Zalvide J, Force T, et al (2011). A novel cardioprotective p38-MAPK/mTOR pathway. Exp. Cell Res., 317:2938-2949. 22001647 10.1016/j.yexcr.2011.09.011 PMC3215777

[133] Birbrair A, Zhang T, Wang ZM, Messi ML, Olson JD, Mintz A, et al (2014). Type-2 pericytes participate in normal and tumoral angiogenesis. Am J Physiol Cell Physiol, 307:C25-38. 24788248 10.1152/ajpcell.00084.2014 PMC4080181

[134] Lemaitre V, Dabo AJ, D’Armiento J (2011). Cigarette smoke components induce matrix metalloproteinase-1 in aortic endothelial cells through inhibition of mTOR signaling. Toxicol Sci, 123:542-549. 21742783 10.1093/toxsci/kfr181 PMC3179676

[135] Xiao Y, Peng H, Hong C, Chen Z, Deng X, Wang A, et al (2017). PDGF Promotes the Warburg Effect in Pulmonary Arterial Smooth Muscle Cells via Activation of the PI3K/AKT/mTOR/HIF-1alpha Signaling Pathway. Cell Physiol Biochem, 42:1603-1613. 28738389 10.1159/000479401

[136] Liu NN, Zhao N, Cai N (2015). Suppression of the proliferation of hypoxia-Induced retinal pigment epithelial cell by rapamycin through the /mTOR/HIF-1alpha/VEGF/ signaling. IUBMB Life, 67:446-452. 25988388 10.1002/iub.1382

[137] Maugeri G, D’Amico AG, Saccone S, Federico C, Cavallaro S, D’Agata V (2017). PACAP and VIP Inhibit HIF-1alpha-Mediated VEGF Expression in a Model of Diabetic Macular Edema. J Cell Physiol, 232:1209-1215. 27661459 10.1002/jcp.25616

[138] Ding Y, Shan L, Nai W, Lin X, Zhou L, Dong X, et al (2018). DEPTOR Deficiency-Mediated mTORc1 Hyperactivation in Vascular Endothelial Cells Promotes Angiogenesis. Cell Physiol Biochem, 46:520-531. 29614494 10.1159/000488619

[139] Pallet N, Thervet E, Timsit MO (2014). Angiogenic response following renal ischemia reperfusion injury: new players. Prog Urol, 24 Suppl 1:S20-25. 24950928 10.1016/S1166-7087(14)70059-4

[140] Zhao H, Huang H, Ologunde R, Lloyd DG, Watts H, Vizcaychipi MP, et al (2015). Xenon Treatment Protects against Remote Lung Injury after Kidney Transplantation in Rats. Anesthesiology, 122:1312-1326. 25856291 10.1097/ALN.0000000000000664

[141] Miller JA, Kirkley KA, Padmanabhan R, Liang LP, Raol YH, Patel M, et al (2014). Repeated exposure to low doses of kainic acid activates nuclear factor kappa B (NF-kappaB) prior to seizure in transgenic NF-kappaB/EGFP reporter mice. Neurotoxicology, 44:39-47. 24813937 10.1016/j.neuro.2014.04.010 PMC4610362

[142] Zhu F, Yue W, Wang Y (2014). The nuclear factor kappa B (NF-kappaB) activation is required for phagocytosis of staphylococcus aureus by RAW 264.7 cells. Exp Cell Res, 327:256-263. 24798100 10.1016/j.yexcr.2014.04.018

[143] Minhajuddin M, Fazal F, Bijli KM, Amin MR, Rahman A (2005). Inhibition of mammalian target of rapamycin potentiates thrombin-induced intercellular adhesion molecule-1 expression by accelerating and stabilizing NF-kappa B activation in endothelial cells. J Immunol, 174:5823-5829. 15843586 10.4049/jimmunol.174.9.5823

[144] Huang WQ, Wen JL, Lin RQ, Wei P, Huang F (2017). Effects of mTOR/NF-kappaB signaling pathway and high thoracic epidural anesthesia on myocardial ischemia-reperfusion injury via autophagy in rats. 10.1002/jcp.26320 29206300

[145] Goncalves GM, Cenedeze MA, Feitoza CQ, Wang PM, Bertocchi AP, Damiao MJ, et al (2006). The role of heme oxygenase 1 in rapamycin-induced renal dysfunction after ischemia and reperfusion injury. Kidney Int, 70:1742-1749. 17003813 10.1038/sj.ki.5001893

[146] Filho DW, Torres MA, Bordin AL, Crezcynski-Pasa TB, Boveris A (2004). Spermatic cord torsion, reactive oxygen and nitrogen species and ischemia-reperfusion injury. Mol Aspects Med, 25:199-210. 15051328 10.1016/j.mam.2004.02.020

[147] Okur MH, Arslan S, Aydogdu B, Zeytun H, Basuguy E, Arslan MS, et al (2017). Protective Effect of Cordycepin on Experimental Testicular Ischemia/Reperfusion Injury in Rats. J Invest Surg: 1-8. 10.1080/08941939.2016.1246629 28402715

[148] Hao M, Zhu S, Hu L, Zhu H, Wu X, Li Q (2017). Myocardial Ischemic Postconditioning Promotes Autophagy against Ischemia Reperfusion Injury via the Activation of the nNOS/AMPK/mTOR Pathway. Int J Mol Sci, 18. 10.3390/ijms18030614 PMC5372630 28287478

[149] Pantazi E, Zaouali MA, Bejaoui M, Folch-Puy E, Ben Abdennebi H, Varela AT, et al (2015). Sirtuin 1 in rat orthotopic liver transplantation: an IGL-1 preservation solution approach. World J Gastroenterol, 21:1765-1774. 25684941 10.3748/wjg.v21.i6.1765 PMC4323452

[150] Wang PR, Wang JS, Zhang C, Song XF, Tian N, Kong LY (2013). Huang-Lian-Jie-Du-Decotion induced protective autophagy against the injury of cerebral ischemia/reperfusion via MAPK-mTOR signaling pathway. J Ethnopharmacol, 149:270-280. 23811213 10.1016/j.jep.2013.06.035

[151] Boengler K, Buechert A, Heinen Y, Roeskes C, Hilfiker-Kleiner D, Heusch G, et al (2008). Cardioprotection by ischemic postconditioning is lost in aged and STAT3-deficient mice. Circ Res, 102:131-135. 17967780 10.1161/CIRCRESAHA.107.164699

[152] Schulman D, Latchman DS, Yellon DM (2001). Effect of aging on the ability of preconditioning to protect rat hearts from ischemia-reperfusion injury. Am J Physiol Heart Circ Physiol, 281:H1630-1636. 11557553 10.1152/ajpheart.2001.281.4.H1630

[153] Boengler K, Schulz R, Heusch G (2009). Loss of cardioprotection with ageing. Cardiovasc Res, 83:247-261. 19176601 10.1093/cvr/cvp033

[154] Murry CE, Jennings RB, Reimer KA (1986). Preconditioning with ischemia: a delay of lethal cell injury in ischemic myocardium. Circulation, 74:1124-1136. 3769170 10.1161/01.cir.74.5.1124

[155] Hausenloy DJ, Yellon DM (2011). The therapeutic potential of ischemic conditioning: an update. Nat Rev Cardiol, 8:619-629. 21691310 10.1038/nrcardio.2011.85

[156] Hausenloy DJ, Mocanu MM, Yellon DM (2004). Cross-talk between the survival kinases during early reperfusion: its contribution to ischemic preconditioning. Cardiovasc Res, 63:305-312. 15249188 10.1016/j.cardiores.2004.04.011

[157] Xuan F, Jian J (2016). Epigallocatechin gallate exerts protective effects against myocardial ischemia/reperfusion injury through the PI3K/Akt pathway-mediated inhibition of apoptosis and the restoration of the autophagic flux. Int J Mol Med, 38:328-336. 27246989 10.3892/ijmm.2016.2615

[158] Aoyagi T, Kusakari Y, Xiao CY, Inouye BT, Takahashi M, Scherrer-Crosbie M, et al (2012). Cardiac mTOR protects the heart against ischemia-reperfusion injury. Am J Physiol Heart Circ Physiol, 303:H75-85. 22561297 10.1152/ajpheart.00241.2012 PMC3404649

[159] Chahine N, Makhlouf H, Duca L, Martiny L, Chahine R (2014). Cardioprotective effect of saffron extracts against acute doxorubicin toxicity in isolated rabbit hearts submitted to ischemia-reperfusion injury. Z Naturforsch C, 69:459-470. 25854766 10.5560/znc.2014-0124

[160] Thoreen CC, Sabatini DM (2009). Rapamycin inhibits mTORC1, but not completely. Autophagy, 5:725-726. 19395872 10.4161/auto.5.5.8504

[161] Yu L, McPhee CK, Zheng L, Mardones GA, Rong Y, Peng J, et al (2010). Termination of autophagy and reformation of lysosomes regulated by mTOR. Nature, 465:942-946. 20526321 10.1038/nature09076 PMC2920749

[162] Zhang D, Contu R, Latronico MV, Zhang J, Rizzi R, Catalucci D, et al (2010). MTORC1 regulates cardiac function and myocyte survival through 4E-BP1 inhibition in mice. J Clin Invest, 120:2805-2816. 20644257 10.1172/JCI43008 PMC2912201

[163] Murakami M, Ichisaka T, Maeda M, Oshiro N, Hara K, Edenhofer F, et al (2004). mTOR is essential for growth and proliferation in early mouse embryos and embryonic stem cells. Mol Cell Biol, 24:6710-6718. 15254238 10.1128/MCB.24.15.6710-6718.2004 PMC444840

[164] Hamacher-Brady A, Brady NR, Gottlieb RA (2006). Enhancing macroautophagy protects against ischemia/reperfusion injury in cardiac myocytes. J Biol Chem, 281:29776-29787. 16882669 10.1074/jbc.M603783200

[165] Paradies G, Paradies V, Ruggiero FM, Petrosillo G (2015). Protective role of melatonin in mitochondrial dysfunction and related disorders. Arch Toxicol, 89:923-939. 25690732 10.1007/s00204-015-1475-z

[166] Heusch G (2015). Molecular basis of cardioprotection: signal transduction in ischemic pre-, post-, and remote conditioning. Circ Res, 116:674-699. 25677517 10.1161/CIRCRESAHA.116.305348

[167] Shih PH, Yen GC (2007). Differential expressions of antioxidant status in aging rats: the role of transcriptional factor Nrf2 and MAPK signaling pathway. Biogerontology, 8:71-80. 16850181 10.1007/s10522-006-9033-y

[168] Heusch G, Boengler K, Schulz R (2010). Inhibition of mitochondrial permeability transition pore opening: the Holy Grail of cardioprotection. Basic Res Cardiol, 105:151-154. 20066536 10.1007/s00395-009-0080-9

[169] Bernardi P, Di Lisa F (2015). The mitochondrial permeability transition pore: molecular nature and role as a target in cardioprotection. J Mol Cell Cardiol, 78:100-106. 25268651 10.1016/j.yjmcc.2014.09.023 PMC4294587

[170] Petronilli V, Miotto G, Canton M, Brini M, Colonna R, Bernardi P, et al (1999). Transient and long-lasting openings of the mitochondrial permeability transition pore can be monitored directly in intact cells by changes in mitochondrial calcein fluorescence. Biophys J, 76:725-734. 9929477 10.1016/S0006-3495(99)77239-5 PMC1300077

[171] Zorov DB, Juhaszova M, Sollott SJ (2014). Mitochondrial reactive oxygen species (ROS) and ROS-induced ROS release. Physiol Rev, 94:909-950. 24987008 10.1152/physrev.00026.2013 PMC4101632

[172] Bernardi P, Petronilli V (1996). The permeability transition pore as a mitochondrial calcium release channel: a critical appraisal. J Bioenerg Biomembr, 28:131-138. 9132411 10.1007/BF02110643

[173] Hausenloy D, Wynne A, Duchen M, Yellon D (2004). Transient mitochondrial permeability transition pore opening mediates preconditioning-induced protection. Circulation, 109:1714-1717. 15066952 10.1161/01.CIR.0000126294.81407.7D

[174] Anzell AR, Maizy R, Przyklenk K, Sanderson TH (2017). Mitochondrial Quality Control and Disease: Insights into Ischemia-Reperfusion Injury. Mol Neurobiol. 10.1007/s12035-017-0503-9 PMC5636654 28401475

[175] Nan J, Zhu W, Rahman MS, Liu M, Li D, Su S, et al (2017). Molecular regulation of mitochondrial dynamics in cardiac disease. Biochim Biophys Acta, 1864:1260-1273. 10.1016/j.bbamcr.2017.03.006 28342806

[176] Oakes SA, Papa FR (2015). The role of endoplasmic reticulum stress in human pathology. Annu Rev Pathol, 10:173-194. 25387057 10.1146/annurev-pathol-012513-104649 PMC5568783

[177] Bronner DN, Abuaita BH, Chen X, Fitzgerald KA, Nunez G, He Y, et al (2015). Endoplasmic Reticulum Stress Activates the Inflammasome via NLRP3- and Caspase-2-Driven Mitochondrial Damage. Immunity, 43:451-462. 26341399 10.1016/j.immuni.2015.08.008 PMC4582788

[178] Watorek E, Szymczak M, Boratynska M, Patrzalek D, Klinger M (2011). Cardiovascular risk in kidney transplant recipients receiving mammalian target of rapamycin inhibitors. Transplant Proc, 43:2967-2969. 21996202 10.1016/j.transproceed.2011.08.009

